# Novel Methods in Disease Biogeography: A Case Study with Heterosporosis

**DOI:** 10.3389/fvets.2017.00105

**Published:** 2017-07-17

**Authors:** Luis E. Escobar, Huijie Qiao, Christine Lee, Nicholas B. D. Phelps

**Affiliations:** ^1^Minnesota Aquatic Invasive Species Research Center, University of Minnesota, St. Paul, MN, United States; ^2^Department of Fisheries, Wildlife, and Conservation Biology, University of Minnesota, St. Paul, MN, United States; ^3^Escuela de Estudios de Postgrado, Facultad de Medicina Veterinaria y Zootecnia, Universidad de San Carlos de Guatemala, Guatemala, Guatemala; ^4^Key Laboratory of Animal Ecology and Conservation Biology, Institute of Zoology, Chinese Academy of Sciences, Beijing, China

**Keywords:** disease biogeography, risk map, ecological niche modeling, minimum-volume ellipsoid, heterosporosis

## Abstract

Disease biogeography is currently a promising field to complement epidemiology, and ecological niche modeling theory and methods are a key component. Therefore, applying the concepts and tools from ecological niche modeling to disease biogeography and epidemiology will provide biologically sound and analytically robust descriptive and predictive analyses of disease distributions. As a case study, we explored the ecologically important fish disease Heterosporosis, a relatively poorly understood disease caused by the intracellular microsporidian parasite *Heterosporis sutherlandae*. We explored two novel ecological niche modeling methods, the minimum-volume ellipsoid (MVE) and the Marble algorithm, which were used to reconstruct the fundamental and the realized ecological niche of *H. sutherlandae*, respectively. Additionally, we assessed how the management of occurrence reports can impact the output of the models. Ecological niche models were able to reconstruct a proxy of the fundamental and realized niche for this aquatic parasite, identifying specific areas suitable for Heterosporosis. We found that the conceptual and methodological advances in ecological niche modeling provide accessible tools to update the current practices of spatial epidemiology. However, careful data curation and a detailed understanding of the algorithm employed are critical for a clear definition of the assumptions implicit in the modeling process and to ensure biologically sound forecasts. In this paper, we show how sensitive MVE is to the input data, while Marble algorithm may provide detailed forecasts with a minimum of parameters. We showed that exploring algorithms of different natures such as environmental clusters, climatic envelopes, and logistic regressions (e.g., Marble, MVE, and Maxent) provide different scenarios of potential distribution. Thus, no single algorithm should be used for disease mapping. Instead, different algorithms should be employed for a more informed and complete understanding of the pathogen or parasite in question.

## Introduction

Disease biogeography is the study of the geographic distribution of infectious diseases (1). It is a powerful approach for mapping disease events, which can inform decision-makers, managers, researchers, and animal and public health specialists (2, 3). Disease biogeography has been proposed as a promising field that can help understand why diseases emerge in one site, but not in another (descriptive analyses), and also provides information to identify suitable areas where outbreaks could occur in the future (predictive analysis) (1).

### Conceptual Bases

According to the assumption of disease biogeography, diseases are not distributed at random across the landscape, instead occur in non-random tractable and quantifiable landscape or environmental conditions. Disease biogeography incorporates the concept of the ecological niche as a crucial element to understand the environmental requirements of a disease transmission system as well as the geographic distribution of the species involved in the system (1, 2). Disease biogeographers use the conceptual bases and methods from the field of ecological niche modeling to make disease biogeography more quantitative (3, 4). Ecological niche modeling links field reports with environmental variables, allowing for development of the descriptive and predictive analyses required by disease biogeography. When ecological niche modeling is used for spatial epidemiology, it varies in complexity, ranging from simple “black-box” approaches (focusing on infected individuals only to reconstruct the conditions where the disease may persist) to more complex hierarchical ecological niche models (including several components of the disease system, e.g., intermediate host, reservoir, vector) (2). Black-box ecological niche models are usually employed for rare diseases where data for susceptible individuals, reservoirs, and vectors is scarce (3). Complex ecological niche models can be developed when more information is available, such as seasonality, density of vectors and reservoirs, and immunity of susceptible hosts, allowing to identify with more detail the different levels of disease transmission risk across areas, periods, and populations (1).

**Figure 1:**
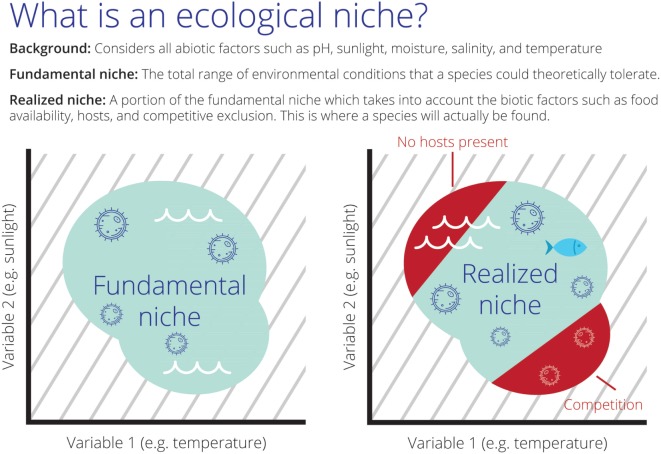
The theoretical scenarios of Fundamental (*N_F_*) and Realized Niches (*N_R_*) of an aquatic parasite in environmental space. Left: all the set of abiotic environmental conditions suitable for the parasite resembling *N_F_* (teal cloud). Right: the sub-set of abiotic environmental conditions suitable for the species resembling *N_R_* (teal cloud). In this scenario, the species is restricted to a portion of *N_F_* due to the effect of biotic interactions (red; e.g., competition with other parasites or absence of fish hosts in the red region making this portion of the niche unusable). Note the background of abiotic environmental conditions available for the species (gray lines) composed by water temperature and sunlight.

Theoretically, species’ niches can be described as Fundamental Niche (*N_F_*) and Realized Niche [*N_R_* (5, 6); Figure 1]. The *N_F_* would resemble the abiotic conditions not modifiable by the species and that are necessary by the species to survive and, most importantly, to maintain populations in the long term without the need for immigration. The *N_R_* is represented by the portion of the *N_F_* that is actually occupied by the species (2). *N_F_* and *N_R_* are usually estimated in ecological niche modeling based on field observations also termed *occurrences* and the environmental conditions in a region, here termed *background*. In the field of ecological niche modeling, considerable efforts have been made to develop methods and environmental variables to determine the *N_F_* and *N_R_* of species under the assumption that *occurrences* ⊆ *N_R_* ⊆ *N_F_* ⊆ *background*. Ecological niche modeling estimations are therefore developed in environmental dimensions to be later projected to geography in the form of maps of areas occupied and potentially occupied by the species in question (Figure 1).

### Applications in Epidemiology

**Figure 2:**
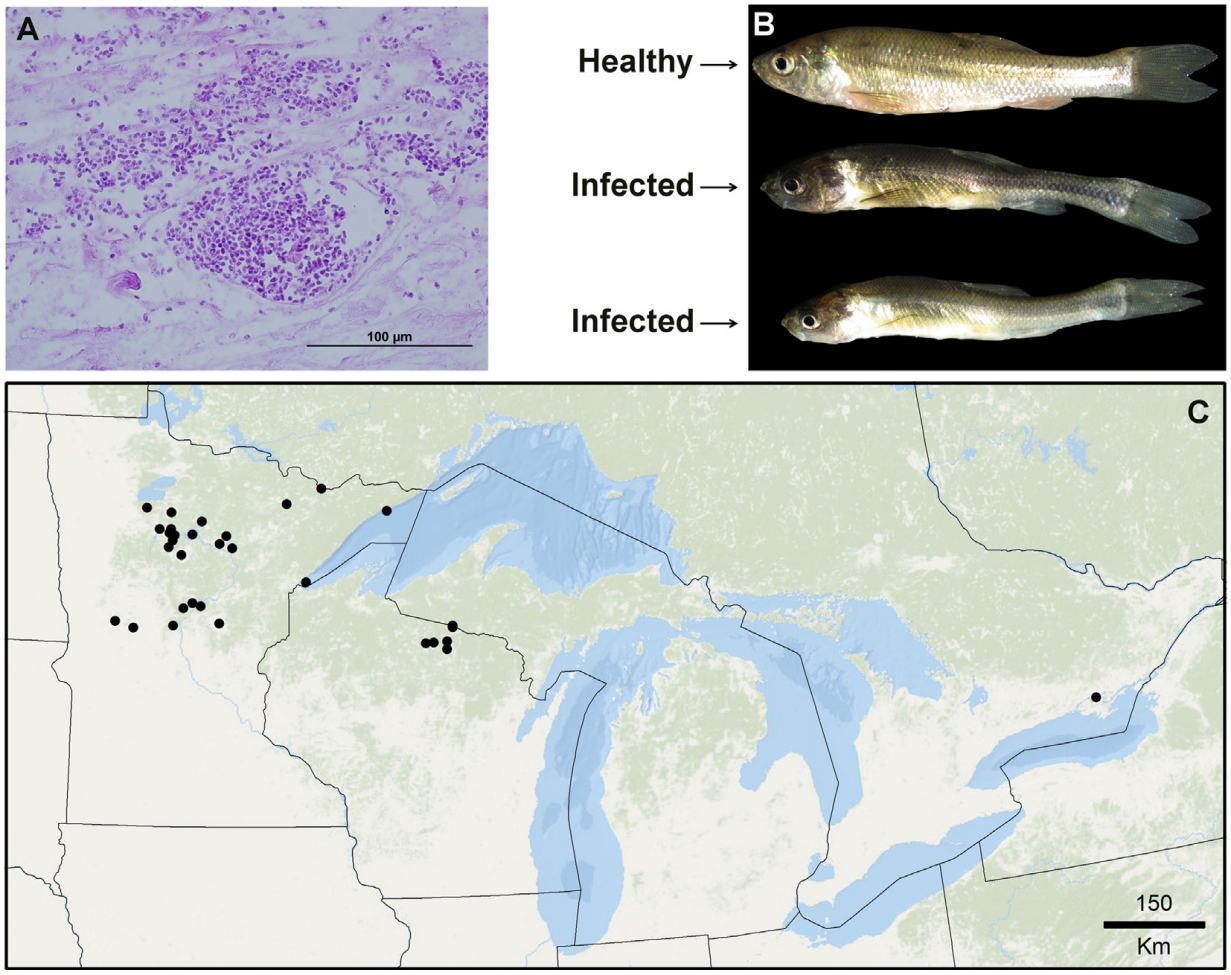
Species used in this exploration. **(A)** Necrotic muscle tissue of the fish Fathead minnows (*Pimephales promelas*) infected with large aggregations of spores from the parasite *Heterosporis sutherlandae*. **(B)** Fathead minnows experimentally challenged with *H. sutherlandae*. **(C)** Heterosporosis-positive occurrences (black points) across the Great Lakes region used for this study. Lines denote administrative boundaries.

While biogeographic methods have gained attention in the epidemiology of terrestrial ecosystems (3), they have been barely explored in the epidemiology of aquatic organisms (7). Examples of biogeographic analyses applied to infectious aquatic diseases include forecasts of *Gyrodactylus salaris* an ectoparasite of salmon (8), *Vibrio cholera* in coastal waters (9), and Viral Hemorrhagic Septicemia virus in the Great Lakes (10). Descriptive biogeographic analyses are useful to understand the natural history of novel infectious diseases, poorly known diseases, or diseases barely explored in the field (11–13). Predictive analyses are useful to anticipate risk in areas where the diseases has not yet been reported, and to guide active surveillance and research (14). A poorly understood infectious disease of epidemiological importance is Heterosporosis which infects fish in the Great Lakes region. Heterosporosis is caused by the microsporidian parasite *Heterosporis sutherlandae* and is known to infect at least eight fish species of economic and ecological importance (15). This disease was first confirmed in 2000 in Leech Lake and Catfish Lake in Minnesota and Wisconsin and has since been reported in waterbodies in Minnesota (*n* = 26), Wisconsin (*n* = 16), Michigan (*n* = 2) in the USA and Lake Ontario (15). The obligate intracellular parasites proliferate inside skeletal muscle cells (Figure 2A), eventually leading to liquefaction of the muscle tissue. Advanced stages of the disease likely result in indirect parasite-induced mortality due to decreased overall fitness, inability to capture prey or escape predation, and increased host stress (Figure 2B). The transmission of *H. sutherlandae* is thought to be horizontal, through the consumption of infected prey or contact with mature spores shed into the water column. Consequently, the overland transport of infected fish or water are likely risk factors for the spread of this pathogen. The possibility does exist for vertical transmission, similar to other microsporidian species infecting fish (16).

With Heterosporosis as a case study, we explored the use of next generation biogeography tools to evaluate how these tools and approaches can help (i) understand the ecology of a rare infectious disease and (ii) forecast the geographic areas where future investigation is necessary. This contribution aims to use the most state-of-the-art algorithms and variables available in order to incorporate disease biogeography in the toolkit of modern epidemiology.

## Methods

### Occurrences

We obtained Heterosporosis-positive occurrence locations from Miller (17) and Phelps et al. (15), who in turn received the reports from natural resource management agencies (i.e., Minnesota Department of Natural Resources, Wisconsin Department of Natural Resources, and U.S. Fish and Wildlife Service). Reports were confirmed by gross lesions and histopathology, and in some cases by PCR and sequencing. Anecdotal reports not verified in the laboratory were not included in this study. Lake centroids were used to determine latitude and longitude locations, and duplicate coordinates were removed. To explore the effect of data curation in the model’s performance, models were developed using all the final occurrences available and a subset of resampled occurrences without environmental outliers (see below).

### Fundamental Niche (*N_F_*)

The *N_F_* was estimated in a large model calibration region including: all the occurrences and the filtered occurrences. Specifically, we focused on the Laurentian Great Lakes region of North America (41.4° and 49.3°N and −97.8° and −74.8°W), a bi-national Canadian–American region with portions of the American states of Ohio, Illinoi, Indiana, Minnesota, Wisconsin, Michigan, Pennsylvania, New York, and the Canadian province of Ontario (Figure 2C). We used climatic variables from this calibration region to construct a *background* of environmental conditions in which the *N_F_* was estimated (18) resembling the landscape and terrestrial environmental drivers where parasites and hosts co-occur. We used climate data from the CliMond repository (19), selecting the first 35 bioclimatic variables with original measurable information on annual, weekly, and seasonal temperature, soil moisture, radiation, and precipitation (Table 1), as these variables are a proxy to reconstruct ecoregions and present-day faunistic distributions (20). These variables are a summary of climatic conditions between 1961 and 1990 in the form of rasters at ~19 km spatial resolution. A principal component analysis was developed using NicheA software 3.0 (21) to reduce dimensionality and correlation between variables, retaining the first three components as they contained 83.85% of the information from the original set of variables. These three components composed the environmental *background* that summarized the environmental patterns in the area with reduced spatial and temporal autocorrelation and were used in posterior analyses. The background developed was then used by the ecological niche model algorithms to identify the relationship of parasite occurrences with this environmental background. Once this relationship is established, models search for this combination of conditions across the entire study area to define locations suitable and unsuitable for the parasite.

**Table 1:** Environmental variables used to construct the background.

Fundamental niche	Realized niche
Annual mean temperature (°C)	Mean value of the monthly MODIS enhanced vegetation index (EVI) time series data (index)
Mean diurnal temperature range [mean(period max-min)] (°C)	SD of the monthly MODIS EVI time series data (index)
Isothermality (Bio02 ÷ Bio07)	Mean value the 8-day MODIS day-time land surface temperature (LST) time series data (°C)
Temperature seasonality (C of V)	SD of the 8-day MODIS day-time LST time series data (°C)
Max temperature of warmest week (°C)	Minimum value of the 8-day MODIS day-time LST time series data (°C)
Min temperature of coldest week (°C)	Maximum value of the 8-day MODIS day-time LST time series data (°C)
Temperature annual range (Bio05-Bio06) (°C)	Mean value the 8-day MODIS night-time LST time series data (°C)
Mean temperature of wettest quarter (°C)	SD of the 8-day MODIS night-time LST time series data (°C)
Mean temperature of driest quarter (°C)	Minimum value of the 8-day MODIS night-time LST time series data (°C)
Mean temperature of warmest quarter (°C)	Maximum value of the 8-day MODIS night-time LST time series data (°C)
Mean temperature of coldest quarter (°C)	Mean value of the 8-day MODIS day-time LST time series data for December/January (°C)
Annual precipitation (mm)	Mean value of the 8-day MODIS day-time LST time series data for February/March (°C)
Precipitation of wettest week (mm)	Mean value of the 8-day MODIS day-time LST time series data for April/May (°C)
Precipitation of driest week (mm)	Mean value of the 8-day MODIS day-time LST time series data for June/July (°C)
Precipitation seasonality (C of V)	Mean value of the 8-day MODIS day-time LST time series data for August/September (°C)
Precipitation of wettest quarter (mm)	Mean value of the 8-day MODIS day-time LST time series data for October/November (°C)
Precipitation of driest quarter (mm)	
Precipitation of warmest quarter (mm)	
Precipitation of coldest quarter (mm)	
Annual mean radiation (W m^−2^)	
Highest weekly radiation (W m^−2^)	
Lowest weekly radiation (W m^−2^)	
Radiation seasonality (C of V)	
Radiation of wettest quarter (W m^−2^)	
Radiation of driest quarter (W m^−2^)	
Radiation of warmest quarter (W m^−2^)	
Radiation of coldest quarter (W m^−2^)	
Annual mean moisture index	
Highest weekly moisture index	
Lowest weekly moisture index	
Moisture index seasonality (C of V)	
Mean moisture index of wettest quarter	
Mean moisture index of driest quarter	
Mean moisture index of warmest quarter	
Mean moisture index of coldest quarter	

*Fundamental niche: variables based on climatic data at ~19 km spatial resolution. Realized Niche: variables based on MODIS data at ~1 km spatial resolution*.

To mitigate uncertainty implicit in occurrences, we employed a method modified from Van Aelst and Rousseeuw (22) as filter to remove potential errors in occurrences. This filtering method is robust for outlier detection: we estimated minimum ellipsoids around occurrences displayed in environmental space and removed 5% [i.e., α = 0.05 (3, 23)] of occurrences with the most marginal environmental values, as these outlier values could be associated with occurrence errors [e.g., misidentification; see, Ref. (24)]. The script for occurrences filtering by detection of the outliers has been included as Supplementary Material S1. We then estimated the *N_F_* using NicheA with the remaining filtered occurrences. The *N_F_* was calculated as the minimum-volume ellipsoid (MVE) from the occurrences in a three-dimensional environmental scenario composed by the first three components from the original environmental variables, described elsewhere (21, 22). Basically, occurrences are displayed and analyzed in three environmental dimensions instead of two geographic dimensions (i.e., latitude and longitude). NicheA estimates the centroid point of the occurrences’ cloud, which will be the center of the ellipsoid. Then, the Euclidean distance is estimated between the center of the ellipsoid and the most external occurrences. The two most external occurrences are the coordinate axes of the ellipsoid and in tandem with the Euclidean distances are used as parameters for a standard tri-axial ellipsoid equation (22). This ellipsoid was then used to simulate Gaussian response curves of the species to the environmental data employed to resemble ecological theories of species responses to environmental conditions (5, 25–27). To visualize the impacts of occurrences curation in estimations, a second model was developed as described above, but without occurrences filtered, i.e., using all the reports available to us.

### Realized Niche (*N_R_*)

The *N_R_* was estimated in a reduced calibration region, including only areas falling inside the *N_F_* model (Figure 1). In these sub-regions, we used 16 remotely sensed variables summarizing land surface temperature (LST) and primary productivity (28). Specifically, we used MODIS data at ~1 km spatial resolution, including day and night-time values of LST, and primary productivity in the form of enhanced vegetation index (EVI; Table 1) available from the WorldGrids repository (28).^1^ These variables were also reduced in number and correlation via a principal component analysis that summarized >89.21% of the overall information from the original variables in the first three components.

We used the Marble algorithm to estimate the *N_R_*. Marble is a novel algorithm that identifies clusters of occurrences in *n-*dimensional environmental spaces as has been described elsewhere (29). Briefly, Marble is based on the generalized density-based clustering algorithm that determines the position of occurrences in the multidimensional environmental space [see, Ref. (30)] and identifies clusters of occurrences of arbitrary shape but also is able to identify noise in the form of non-clustered occurrences in the environmental space [see, Figure 6 in Ref. (29)]. The default parameters are the automatic estimation of the radii according to the number and position of occurrences allowing the inclusion of at least 99% of occurrences in the clusters. Due to the ability of the Marble algorithm to prioritize groups of occurrences and exclude isolated occurrences, the algorithm generates ecological niche models from consistent clusters only, with reduced interpolation and extrapolation. This approach results in models of metamorphosed shapes in the environmental space (29). The script employed in this study to develop Marble models in R has been included as Supplementary Material S2. We employed the occurrences and MODIS data that were inside the areas predicted by the *N_F_* model. The *N_F_* and *N_R_* were then projected to the geographic space to identify areas suitable as predicted by the models.

Finally, to highlight the predictions of MVE and Marble vs. a classic ecological niche modeling method, we developed a series of models using Maxent algorithm (32). Maxent is a type of logistic regression (33) and is currently a standard method to estimate species’ ecological niches (34). Maxent models included the estimation of the *N_F_* based on climate data and *N_R_* based on remote sensing data. The *N_F_* and *N_R_* were estimated using the original occurrences and filtered occurrences as described before. Models were calibrated using default settings in Maxent 3.3.3k (34).

All models were compared using a cumulative binomial distribution test using two sets of occurrences, one for model calibration and one for model evaluation, as in Peterson et al. (24). The R script used here for automated data split is included as Supplementary Material S3. Evaluation occurrences were not used during model calibration and instead were used to test the ability of the model to predict independent data using evaluation points as trials, evaluation points predicted correctly as successes, and the proportion of area predicted suitable as the probability of a success (23). The method used to develop this evaluation is included as Supplementary Material S4 to facilitate replication.

## Results

**Figure 3:**
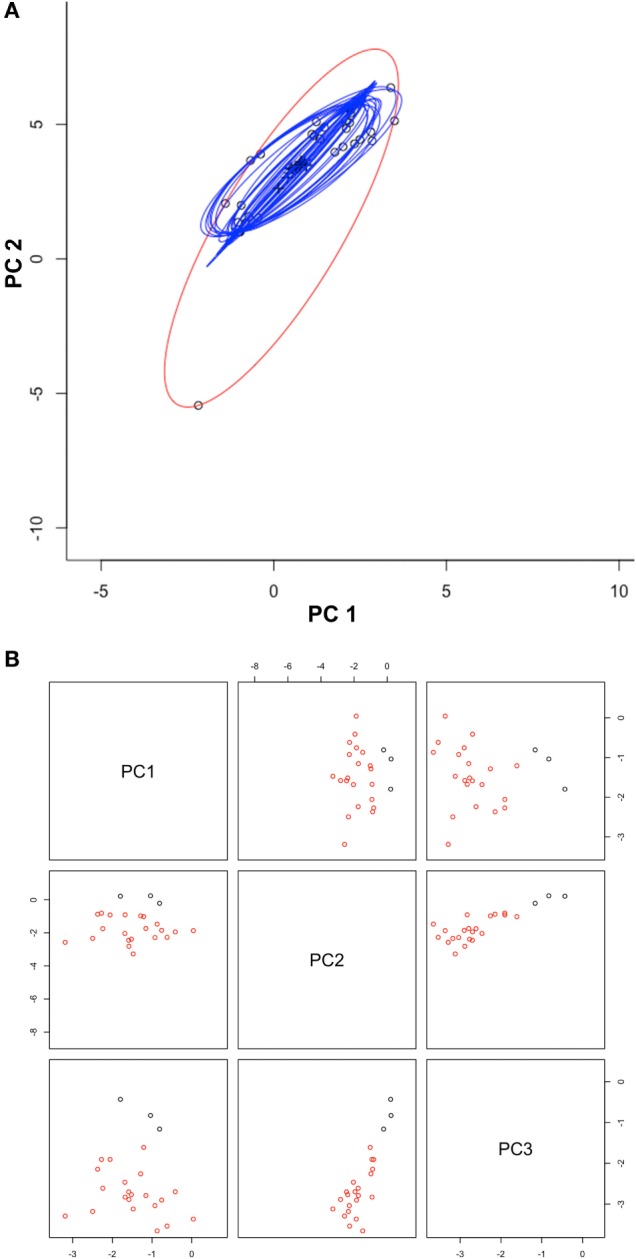
Automated occurrences curation process. **(A)** Occurrences (black circles) were displayed in a two-dimensional environmental space of principal components one (PC1) and two (PC2) from the original climate data. Ellipsoids were estimated using the full occurrences (red ellipsoid) and then reducing one occurrence at a time (blue ellipsoids), to filter occurrences via outlier elimination. Note that using 100% of the points resulted in the detection of an outlier (black circle in edge of the red ellipsoid). **(B)** The first three PC from MODIS data were used to display the distribution of filtered occurrences (red circles) and also occurrences detected clusters (black circles). Note that outlier occurrences in term of climate were also outliers in terms of MODIS data (black points). The script for outlier detection is included as Supplementary Material S1.

**Figure 4:**
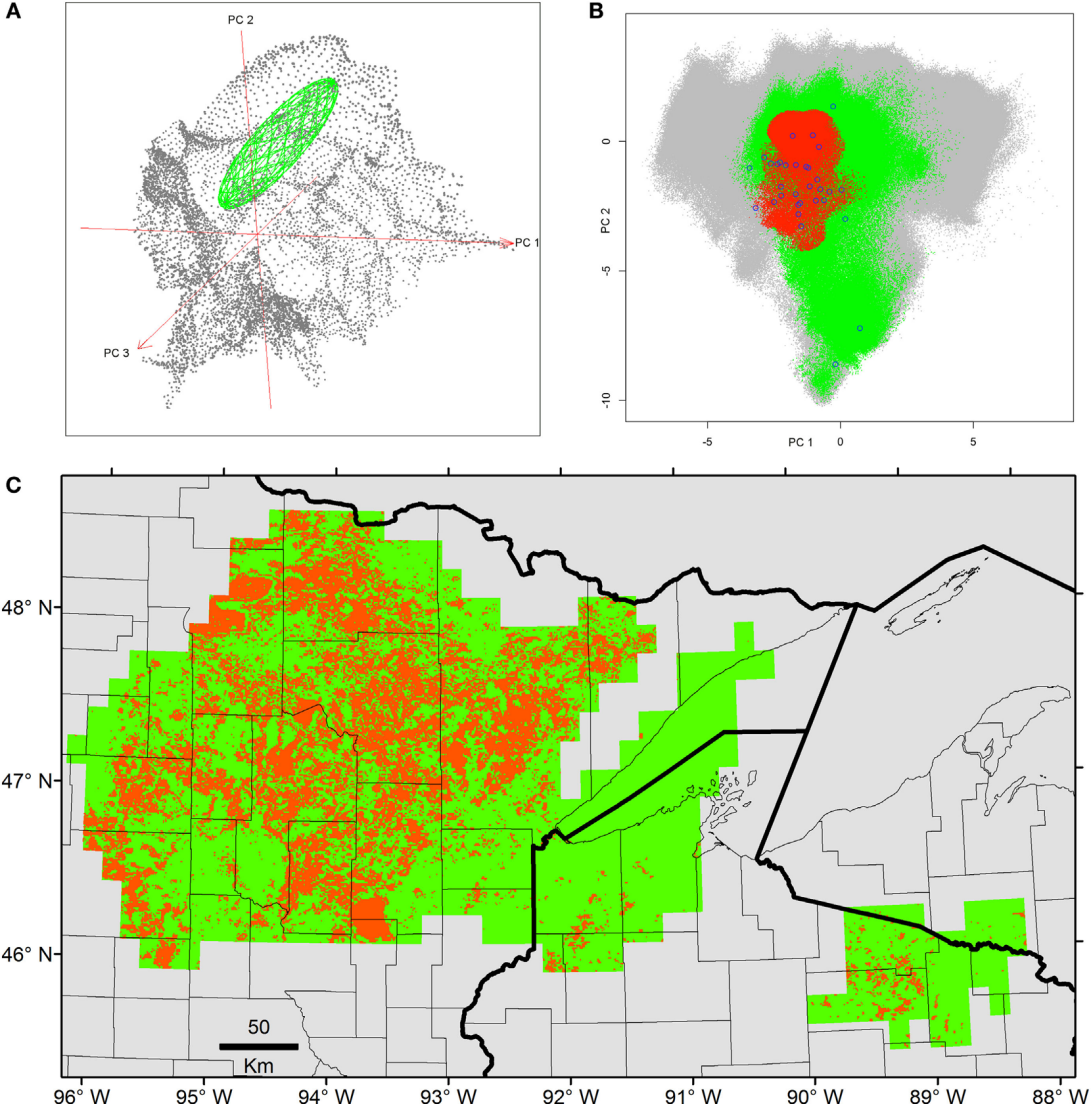
Ecological niche models from filtered Heterosporosis data. **(A)** The Fundamental Niche (*N_F_*; green ellipsoid) was estimated based on the minimum-volume ellipsoid formula in NicheA, using as background (gray points) the first three principal components (PC) of climate (red axes). **(B)** The Realized Niche (*N_R_*; red) as estimated inside the conditions predicted suitable by the *N_F_* (green) across the *background* constructed with the PC of the MODIS data (gray). **(C)** The *N_F_* (green) and the *N_R_* (red) were projected to the geography. In this case, the axes are longitude and latitude.

**Figure 5:**
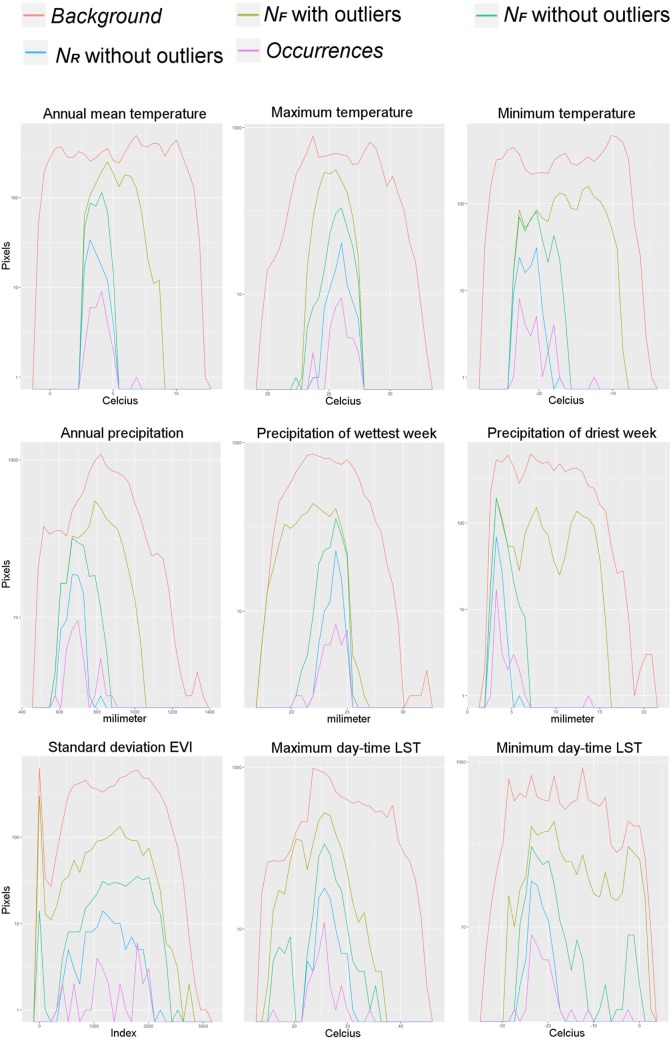

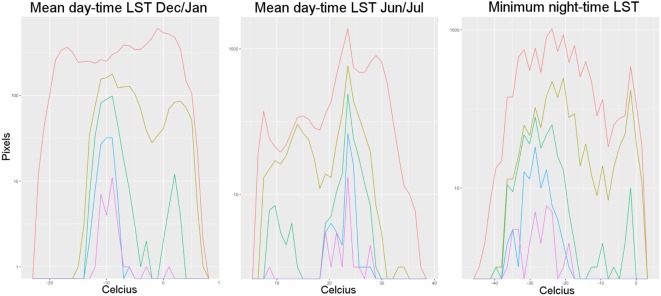
Example of predictions represented in terms of single environmental variables. Pixels values of each environmental variable were counted across the study area representing the *background* (red line), the pixels predicted suitable by the Fundamental Niche (*N_F_*) models based on a minimum-volume ellipsoid including all occurrences, i.e., with the environmental outlier occurrences (olive line), and with outliers removed (green line), and the estimation of environments occupied as predicted by the Realized Niche (*N_R_*) model from the Marble algorithm (blue line). The *occurrences* employed for model calibration are also displayed (pink line). Count of pixels in log value for better visualization. Note that including all the occurrences without filtering generates high extrapolation of the *N_F_* (i.e., broader range from the *N_F_* estimations; olive line) compared with the models based on filtered occurrences (i.e., green line).

Once duplicates and environmental outliers were removed, 32 single occurrences remained and were used for modeling. The data curation process in the environmental space allowed us to identify several environmental outlier occurrences; one was removed based on our threshold defined *a priori* (Figure 3). The MVE estimated from this set of filtered occurrences, as a proxy of the *N_F_*, revealed that the species was not occurring in all environmental conditions available in the model calibration region, instead, it occurred in consistent, tractable climatic conditions (Figures 4 and 5). When the *N_F_* was projected from the environmental space to the geographic space, suitable areas were identified across North central Minnesota, northern areas of Wisconsin, and a small portion of western Michigan (Figure 4). Once the *N_R_* of the parasite was estimated in these areas, we found suitability in specific areas of these states with high detail that allowed the identification of lakes that could be suitable for Heterosporosis (Figure 4). The Marble algorithm estimated fine scale suitability as a proxy of the *N_R_*, based on a cloud of occurrences that excluded three isolated marginal occurrences detected outside of a main cluster (Figure 3). This generated a model of suitability based on the occurrences occupying the most tractable and consistent environmental conditions.

**Figure 6:**
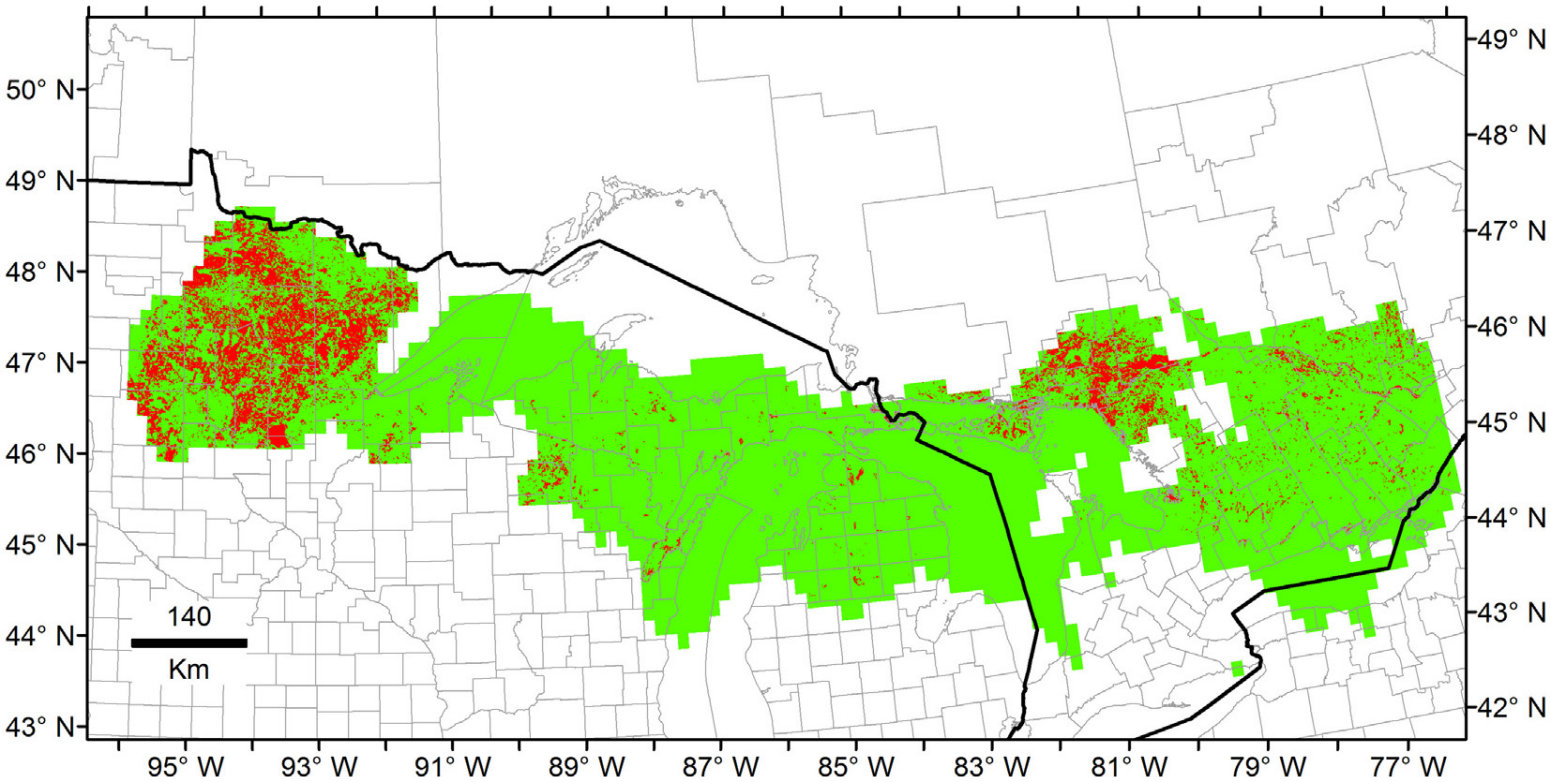
Example of ecological niche models from unfiltered Heterosporosis data. The Fundamental Niche (*N_F_*; green) was estimated based on the minimum-volume ellipsoid formula in NicheA, using all the occurrences including outliers and, as background, the first three principal components (PC) of climate. Then, the Realized Niche (*N_R_*; red) was estimated inside the Fundamental Niche using marble algorithm based on the PC of the MODIS data (similar to Figure 4 but with unfiltered occurrences).

Once models were calibrated using all the data available, including the climatic outlier (Figure 3), the ecological niche models predicted broader areas suitable for Heterosporosis across the Great Lakes basin, resulting in 406% increase in areas predicted for this *N_F_* model compared with the *N_F_* without outliers (Figure 6). Changes in *N_F_* estimations generated changes in the range of environmental values predicted suitable for the parasite (Figure 5). Changes in the range of environmental tolerances occurred in the highest limit for some variables, while others showed shifts in the lowest limits. For some variables (e.g., maximum temperature, precipitation of wettest week, SD EVI, and maximum day-time LST), the impact of the outlier in the range of environmental tolerances was minimal, while others had more dramatic impacts in the range estimated (e.g., annual mean and minimum temperature, annual precipitation, and precipitation of the driest week; Figure 5).

**Figure 7:**
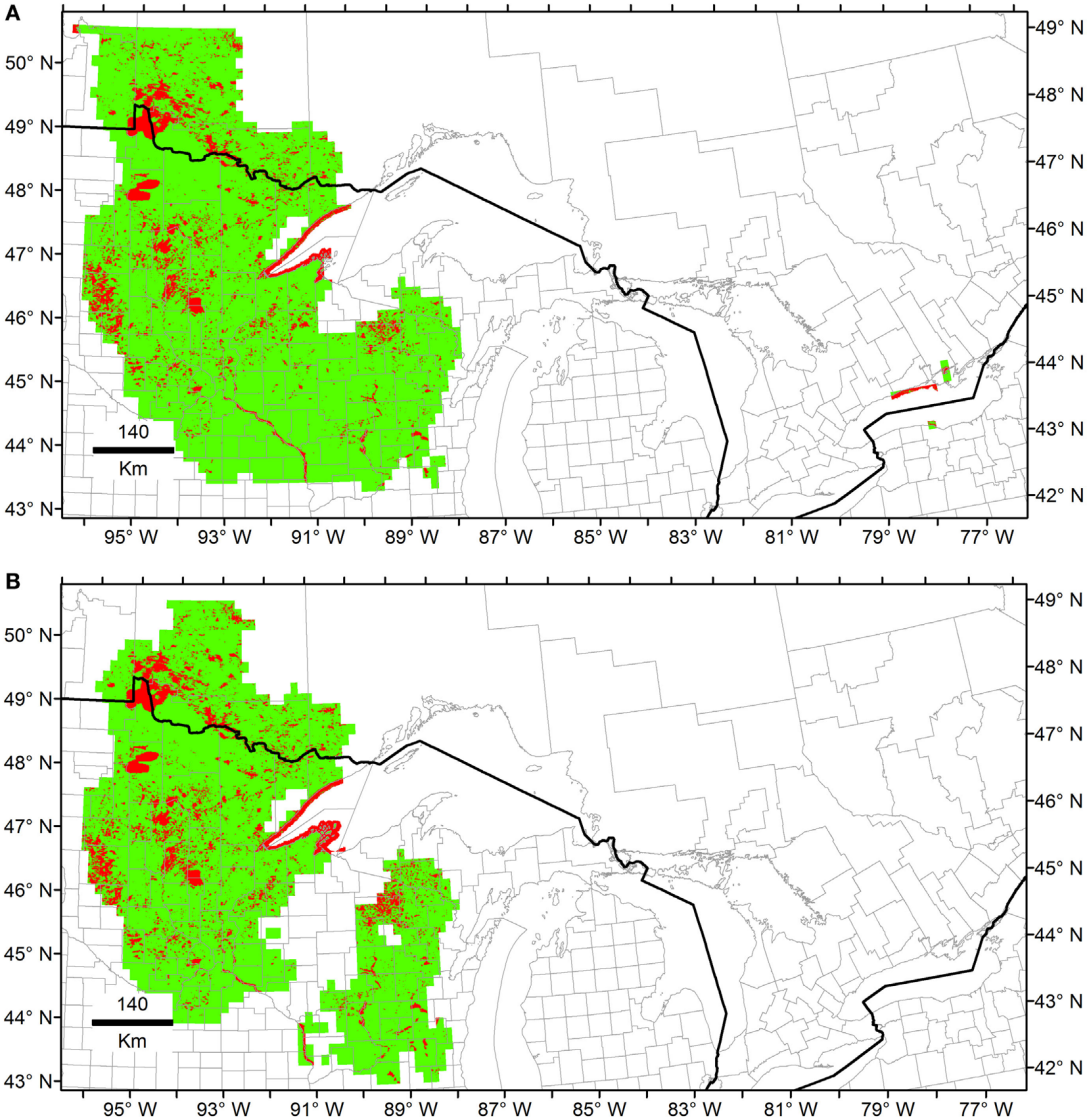
Ecological niche models from Heterosporosis data using Maxent. The Fundamental Niche (*N_F_*; green) was estimated using as background the first three principal components (PC) of climate. Then, the Realized Niche (*N_R_*; red) was estimated inside the Fundamental Niche based on the PC of the MODIS data. **(A)** Models using all the occurrences available. **(B)** Models based on filtered data without outliers.

Maxent models generated predictions comparable to those of Marble in the regions of Minnesota and Wisconsin. However, Maxent predictions were restricted to areas surrounding the occurrences when the entire data set was employed, showing low effect of outliers during model calibration as compared to MVE models (Figure 6 vs. Figure 7). Using independent calibration and evaluation occurrences during model evaluations, all models showed prediction better than by chance in all the scenarios (Supplementary Material S5). The outputs, however, varied between algorithms. For example, we found that estimations of *N_F_* was overfitted in Maxent, while MVE provided more generalized predictions when the model was calibrated using all the data available (Figure 6 vs. Figure 7A).

## Discussion

Ecological niche models for Heterosporosis allowed the identification of suitable areas beyond the current locations with reports of the parasite, providing information about sites where the parasite could potentially occur based on suitable environmental conditions (4). MVE and Marble, the two novel algorithms employed in the modeling process, generated suitability surfaces in the form of binary maps showing areas with environmental conditions similar to those with Heterosporosis records (Figures 4 and 6). This binary modeling output format avoids continuous suitability surfaces of difficult biological interpretation (3). The models based on filtered occurrences without environmental outliers generated models with the best fit as expressed by the similarity of environmental conditions occupied by the occurrences vs. the conditions predicted by the MVE. That is to say, failure to remove outlier occurrences may have severe consequences in the areas predicted suitable by some ecological niche model algorithms (35), including MVE (see Figure 4 vs. Figure 6). For example, removing outlier occurrences generated models with more detailed identification of regions suitable for Heterosporosis, thus, making forecasts a more useful tool to guide active epidemiological surveillance in specific constrained areas.

We found that the inclusion of environmental outliers also had a dramatic impact on the predictions in both the geographic and the environmental space. In this case, this was particularly true for the *N_F_* models based on the MVE algorithm. For example, models calibrated with the environmental outlier generated predictions with high extrapolation for the higher values of predicted suitability, including annual mean and minimum temperature and annual precipitation and precipitation of driest week. For other variables, such as precipitation of wettest week, the outlier generated extrapolation in the lower values (Figure 5). We found, however, that in other variables the inclusion or not of the outlier occurrence was less dramatic (e.g., maximum temperature, SD of EVI, day-time LST values for the annual maximum and minimum, and the mean values for December and January, and for June and July; Figure 5). The Marble algorithm was less sensitive since this method automatically accounts for occurrences outside environmental clusters (Figure 3), i.e., noise detection (30).

### Fundamental Niche (*N_F_*)

According to ecological theories, the *N_F_* of an organism should have an ellipsoidal form (21). This assumption is supported by experimental data showing Gaussian responses of species to abiotic environmental variables (26, 27, 36–39). The MVE estimated from the occurrences in environmental dimensions was able to generate response curves resembling normal distributions as the theory suggested (Figure 5), allowing us to have a proxy of the environmental tolerances of the species according to the data available to us. This suggests that NicheA could be a promising tool to simulate how species occupy environmental conditions based on field records; however, this would require high quality records. Erroneous records could tremendously impact the range of values used to estimate the ellipsoids (30), and in turn, the areas predicted suitable (Figure 5). To mitigate the inclusion of errors from the set of occurrences (40), we propose to employ an automated data curation system developed in environmental dimensions (Figure 3).

In addition to occurrence filtering, the estimation of MVEs is a protocol that requires a series of steps including a PCA analysis, displaying occurrences in the environmental space, calculations of ellipsoids, and projection of the final model to the geographic space. To facilitate this process, the workflow of the analyses developed here is included as Supplementary Material S6 to be executed in NicheA (21) and includes data to replicate this workflow (Supplementary Material S6). Step-by-step instructions to estimate *N_F_* of any species can also be found in the website of NicheA.^2^

### Realized Niche (*N_R_*)

While the *N_F_* aims to estimate environmental tolerances, algorithms to estimate *N_R_*, as the case with Marble, are meant to identify in environmental space the most “immediate” environmental conditions that are suitable to the species. In other words, models aiming to estimate the *N_R_* are expected to overfit to the occurrences used for model calibration, resulting in a reduced interpolation and extrapolation. To our knowledge, this is the first application of Marble in epidemiology, and in turn in modeling diseases in fish. We showed that Marble is a promising algorithm to estimate realized niches, which in turn estimates areas that are suitable in high detail, avoiding the inclusion of environmental conditions beyond those currently used by the species.

### Novel vs. Classic Methods

We explored two novel methods to estimate species niches based on (i) algorithms resembling ecological theories (i.e., MVE and Marble) and (ii) algorithms resembling the data (i.e., Maxent). All models showed that predictions of independent occurrences were better than random in all model scenarios. However, it was evident that the machine learning structure of Maxent provides a high fit of the model with the data available (33). If assumptions are more relaxed and the data and information of the species are limited, MVE can be a good solution as this algorithm is less complex than Maxent (requires less parameters during calibration). This predictive behavior was replicated during *N_R_* estimations: Marble provided generalized estimations with broad areas predicted suitable for the parasite and Maxent provided more conservative estimations principally in sites surrounding reports. We note that both modeling approaches, (i) algorithms resembling ecological theories (i.e., MVE and Marble) and (ii) algorithms resembling the data, are not wrong. In fact, both approaches develop niche estimations based on different assumptions: algorithms resembling ecological theories may overestimate the areas suitable due to the high levels of interpolation (31) aiming to reconstruct niche shapes as supported species physiology (21), while machine learning algorithms may have increased sensitivity to the data due to reduced extrapolation and interpolation to gain model fit. We argue that both approaches have pros and cons, one can prefer a simple model generalizing the niche estimation to gain knowledge or one can prefer a model with limited overestimation to obtain predictions dictated by the data. Under both scenarios, the study question and assumptions will vary. For example, one can assume that Heterosporosis is still on its path to occupy the full ecological niche (i.e., ecological equilibrium) and model over estimations reducing the overfit of models to the data would be desirable. To mitigate uncertainties during model selection, two main frameworks could be considered in ecological niche modeling, one in which several algorithms are explored to capture consensus and variability (31), and one in which a single algorithm is explored under a detailed parameterization and assumptions based on abundant data and a considerable knowledge of the species in question (41).

### Further Research

Current methods for disease mapping in epidemiology are dominated by distance-based analyses restricted to geography (e.g., spatial clusters), neglecting the importance of the landscape heterogeneity (42). However, recent literature in epidemiology has attempted to consider the climate and/or the landscape configuration when mapping disease transmission risk (1). While these attempts have important benefits in terms of the information generated and biological realism in the maps produced, most of these studies still lack a biogeographic framework to design the study and interpret the results. Indeed, click-and-run tools to generate ecological niche models are common in the scientific literature with studies of poor study design, but more strikingly without justification of the model parameters, assumptions, variables, occurrences, and study areas selected, even when such factors have been largely recognized as crucial in ecological niche modeling (4, 33–35, 43, 44).

Our study case focused on a fish parasite; thus, the model was calibrated using exclusively infected fish, resulting in a “black-box” approach as a proxy for all the species acting in the Heterosporosis system: the parasite and the susceptible hosts (2). Future studies are necessary at finer scales in the areas identified here as suitable for the parasite to include fish density, fish community assemblages, and other competitive parasites limiting the occurrence of Heterosporosis at a local level.

We assumed that *N_F_* could be reconstructed using environmental data at coarse resolution, while *N_R_* would require environmental variables at finer grain. These assumptions may be a limitation to the areas predicted by the models and should be a crucial point during the study design of models developed for spatial epidemiology. Beyond resolution, models could be impacted by the assumptions on the response of species to the environmental values absent in the occurrence data available. An important assumption is environmental interpolation. MVE has high interpolation of values predicting suitable all the environmental conditions falling inside the range of values estimated from the available occurrences. Thus, MVE would be less sensitive to sampling bias but would be sensitive to outliers. Maxent and Marble have limited interpolation with overfit to the data available, resulting in suitable conditions resembling the data. Thus, these algorithms are more sensitive to sampling bias (e.g., oversampling close to the roads or only during summer conditions) but are less sensitive to outliers. A good practice would be a careful selection of algorithms with the abilities to answer the research question, i.e., estimation of the potential distribution (*N_F_*) or current distribution of the disease (*N_R_*), considering the weaknesses in the environmental data (e.g., resolution) and occurrence data (e.g., bias).

### Final Remarks

Several ecological niche modeling tools exist to map infectious diseases, but easy-to-use tools are preferred even if most users do not understand how the algorithms work (45). For instance, Maxent, an easy-to-use ecological niche modeling software, has suffered abuse in its application to epidemiology in a series of “recipe-like” studies with Maxent assumptions that may not be appropriated to the particular study questions (1, 3, 46–48). In biogeography, ecological niche modelers have cautioned the development of models with poor study design (3, 40, 46, 49, 50), which may lead to incorrect assumptions and interpretations. The algorithm selection and study design is particularly crucial in applications of ecological niche modeling to epidemiology, considering that modeling outputs could be used by public health intelligence and animal health policy makers.

We propose novel ecological niche modeling methods that can help understand the biogeography of an aquatic infectious disease, identify areas at risk for disease transmission, and can complement current methods. First, we highlight the importance of data curation and show a method for outlier removal in environmental dimensions based on *a priori* assumptions. Also, the ecological niche modeling algorithms proposed require low parameterization as they are based on the position (MVE) and density (Marble) of occurrences in an environmental space (22, 30), but also require a series of biological assumptions to make the outputs interpretable [e.g., Fundamental Niches of an ellipsoidal shape (21)]. We found that exploring algorithms of different analytical nature such as those aiming to fit environmental clusters, climatic envelopes, and logistic regressions (e.g., Marble, MVE, and Maxent) provided different scenarios of the potential distribution of Heterosporosis. Thus, no single algorithm should be used for disease mapping as this may result in an incomplete panorama of forecasts. We argue that different algorithms are necessary to achieve more informed predictions of the potential distribution of pathogen or parasites of public health or veterinary concern.

## Author Contributions

LE conceived and designed the study, collected and analyzed the data, and wrote the paper. HQ analyzed the data and co-wrote the paper. CL co-wrote the paper. NP collected the data and co-wrote the paper. All authors approved the final version of this manuscript.

## Conflict of Interest Statement

The authors declare that the research was conducted in the absence of any commercial or financial relationships that could be construed as a potential conflict of interest.

## Footnotes

^1^http://worldgrids.org.

^2^http://nichea.sourceforge.net/.

## Supplementary Material

Supplementary Material S1 R script for outliers detection in environmental space (DOCX).

Supplementary Material S2 R script to develop ecological niche models using Marble (DOCX).

Supplementary Material S3 R script to split the occurrence data for model evaluation as in Ref. (24) (TXT).

Supplementary Material S4 Spreadsheet formula to develop model evaluation (XLSX).

Supplementary Material S5 Model evaluation results (DOCX).

Supplementary Material S6 NicheA workflow and data to replicate analyses (XML).
