# Supplementary material for: Novel Methods in Disease Biogeography: A Case Study with Heterosporosis
Source: Front Vet Sci. 2017 Jul 17;4:105. doi: 10.3389/fvets.2017.00105 (PMC5511963; doi:10.3389/fvets.2017.00105)
Supplement: Supplementary Material S1 — R script for outliers detection in environmental space (DOCX). [file data_sheet_1.pdf]

Supplementary Material

Novel Methods in Disease Biogeography:

A Case Study with Heterosporosis

Luis E. Escobar*, Huijie Qiao, Christine Lee, Nicholas B. D. Phelps

*** Correspondence:** Corresponding Author: lescobar@umn.edu

1 Supplementary Material S1

```
#Marble script

require(dbscan)

require(geometry)

require(raster)

#Set up working directory

setwd("/Users/huijieqiao/Experiments/Heterosporis/")

#Load required environmental layers

raster_modis<-list.files("RS_cut")

raster_modis<-c("PC1.tif", "PC2.tif", "PC3.tif")


#Set up mask layer

mask<-raster("PCA_RS_cut/PC1.tif")

not_na<-!is.na(values(mask))


#Load occurrences

d<-read.table("HTSP_ll.csv", head=T, sep=",")
```

```
#Set up the basical folder name which is used to save the results
base_name<-"MA"

sample<-d
test<-data.frame(v=values(mask))

#Extract the values of occurrences from environmental layers
for (modis in raster_modis){
  r_modis<-raster(paste("PCA_RS_cut/",
                        modis, sep=""))

  sample[,gsub(".tif", "", modis)]<-extract(r_modis, d)
  test[,gsub(".tif", "", modis)]<-values(r_modis)

}

sample<-sample[, c(3:ncol(sample))]

#Estimate the parameters of MA: eps and minPts
eps<-quantile(dist(sample[,]), 0.1)[1]
min_point<-trunc(nrow(sample) * .1)

#Build the dbscan (MA) model
ma <- dbscan(sample, eps = eps, minPts = min_point)
```

```
#Export result
png(filename = paste(base_name, "/model.png", sep=""),
    width = 8, height = 8, units = "in", pointsize = 12,
    bg = "white",  res = 300)
plot(sample, col=ma$cluster)
points(sample[ma$cluster==0,], pch = 3, col = "grey")
dev.off()

#Invoke model to the test set.
test<-test[which(not_na),]
test<-test[ , -which(names(test) %in% c("v"))]
test_o<-test
test<-test[complete.cases(test),]
test_result<-test
test_result$ma_result<-0
for (i in seq(1, nrow(test), by=10000)){
  print(paste(i, nrow(test), sep="/"))
  if ((i+10000)>nrow(test)){
    test_t<-test[c(i: nrow(test)), ]
  }else{
    test_t<-test[c(i: (i+10000 - 1)), ]
  }

  ma_result<-predict(ma, sample, test_t)
  if ((i+10000)>nrow(test)){
```

```
    test_result[c(i: nrow(test)),]$ma_result<-ma_result

  }else{

    test_result[c(i: (i+10000 - 1)),]$ma_result<-ma_result

  }

}




#Export result

write.table(test_result, paste(base_name, "/ma.csv", sep=""), row.names =
F, sep=",")




png(filename = paste(base_name, "/ma.png", sep=""),

    width = 8, height = 8, units = "in", pointsize = 12,

    bg = "white",  res = 300)

colors<-rainbow(length(unique(ma$cluster))-1)


plot(test_result$PC1, test_result$PC2, pch=".", col="grey")

points(test_result[which(test_result$ma_result!=0), ]$PC1,

       test_result[which(test_result$ma_result!=0), ]$PC2, pch=".",
col=colors[test_result$ma_result])

points(sample$PC1, sample$PC2, col="blue")

dev.off()


value<-values(mask)
```

```
value[which(not_na & complete.cases(test_o))]<-test_result$ma_result
value[which(value>0)]<-1

values(mask)<-value
writeRaster(mask, filename=paste(base_name, "/MA.tif", sep=""),
            format="GTiff", overwrite=T)
```
