# Supplementary material for: Novel Methods in Disease Biogeography: A Case Study with Heterosporosis
Source: Front Vet Sci. 2017 Jul 17;4:105. doi: 10.3389/fvets.2017.00105 (PMC5511963; doi:10.3389/fvets.2017.00105)
Supplement: Supplementary Material S2 — R script to develop ecological niche models using Marble (DOCX). [file data_sheet_2.pdf]

2 Supplementary Material S2

#Detect outliers

```
#Load functions

source("functions.R")


#Load environmental layers

environment_layers<-
paste("/Users/huijieqiao/Experiments/Heterosporis/current_10min_cut/",


list.files("/Users/huijieqiao/Experiments/Heterosporis/current_10min_cut/"
),

                          sep="")


#Load occurrences

occ<-read.table("HTSP_ll.csv", head=T, sep=",")



#Set up parameters

is_pca<-T

first_pcs<--1

proportion_threshold<-0.85

max_removed_occurrences<-10000

tolerance<-0.01


#Detect outliers

DETECT_OUTLIER(occ, environment_layers, max_removed_occurrences=1)
```

```r
#Export results
save(mve_definitions, vol_list, file = "result.RData")
```

function.R

```r
require(geometry)
library(raster)
library(rgdal)
library(dplyr)
##begin of functions

#Get the definition of MVE
MVE_DEFINITION<-function(samples, tolerance = 0.01){
  mve<-MinVolEllipse(samples, tolerance)
  return (mve)
}

#Test the point is inside of a given MVE or not
IN_MVE<-function(all_samples, definition, dimension){

  mve_a<-definition[[1]]
  mve_c<-definition[[2]]
  sub_d<-as.matrix(all_samples)
  result<-logical(length(sub_d)/dimension)
  for (i in 1:(length(sub_d)/dimension)){
```

```
    x<-as.matrix(sub_d[i,])
    distance <- t(x-mve_c)%*%mve_a%*%(x-mve_c)
    if ((distance[1]-1)<=0.001){
      result[i] <- TRUE
    }else{
      result[i] <- FALSE
    }
  }
  return(result)
}

#Define the %^% symbol
"%^%" <- function(S, power)
  with(eigen(S), vectors %*% (values^power * t(vectors)))

#Generate MVE
MinVolEllipse<-function(P, tolerance){
  ##P<-true_set
  ch <- convhulln(P)
  vex <- unique(as.integer(ch))
  P<-P[vex,]
  P<-t(as.matrix(P))

  d<-dim(P)[1]
  N<-dim(P)[2]
```

```
  Q<-matrix(0,nr=d+1,nc=N)
  Q[1:d,]<-P[1:d,1:N]
  Q[1+d,]<-1
  count<-err<-1
  u<-rep(1/N,N)
  while(err>tolerance){
    X<-Q%*%diag(u,N,N)%*%t(Q)
    M<-diag(t(Q)%*%solve(X)%*%Q)
    maxM<-max(M)
    j<-order(M)[N]
    step.size<-(maxM-d-1)/((d+1)*(maxM-1))
    new.u<-(1-step.size)*u
    new.u[j]<-new.u[j]+step.size
    count<-count+1
    ll<-svd(new.u-u)
    err<-max(ll[[1]])
    u<-new.u
  }
  U<-diag(u,N,N)
  A<-solve(P%*%U%*%t(P)-(P%*%u)%*%t(P%*%u))/d
  c<-P%*%u
  return(list(A,c))
}

#Calculate the volume of a given ellipsoid
VOLUME_MVE<-function(definition){
```

```
  mve_a<-definition[[1]]
  mve_c<-definition[[2]]
  volume<-(4/3) * pi * sqrt(det(mve_a%^%-1))
  return(volume)
}



ELLIPSEM <-
  function (mu, amat, c2, npoints = 100, showcentre = T, ...)
  {
    if (all(dim(amat) == c(2, 2))) {
      eamat <- eigen(amat)
      hlen <- sqrt(c2/eamat$val)
      theta <- angle(eamat$vec[1, 1], eamat$vec[2, 1])
      ellipse(hlen[1], hlen[2], theta, mu[1], mu[2], npoints = npoints,
              ...)
      if (showcentre)
        points(mu[1], mu[2], pch = 3)
    }
    invisible()
  }

ellipse <-
  function (hlaxa = 1, hlaxb = 1, theta = 0, xc = 0, yc = 0, newplot = F,
            npoints = 100, ...)
```

```
  {
    a <- seq(0, 2 * pi, length = npoints + 1)
    x <- hlaxa * cos(a)
    y <- hlaxb * sin(a)
    alpha <- angle(x, y)
    rad <- sqrt(x^2 + y^2)
    xp <- rad * cos(alpha + theta) + xc
    yp <- rad * sin(alpha + theta) + yc
    if (newplot)
      plot(xp, yp, type = "l", ...)
    else lines(xp, yp, ...)
    invisible()
  }
angle <-
  function (x, y)
  {
    angle2 <- function(xy) {
      x <- xy[1]
      y <- xy[2]
      if (x > 0) {
        atan(y/x)
      }
      else {
        if (x < 0 & y != 0) {
          atan(y/x) + sign(y) * pi
        }
```

```
        else {
          if (x < 0 & y == 0) {
            pi
          }
          else {
            if (y != 0) {
              (sign(y) * pi)/2
            }
            else {
              NA
            }
          }
        }
      }
    }
    apply(cbind(x, y), 1, angle2)
  }
##end of functions

DETECT_OUTLIER<-function(occ, environment_layers,
                         is_pca=T, first_pcs=3, proportion_threshold=0.85,
                         max_removed_occurrences=100, tolerance = 0.01){
  ##begin of the function
  #read all the raster files
  for (i in c(1:length(environment_layers))){
```

```
  print(paste("Reading raster file", environment_layers[i]))
  r <- raster(environment_layers[i])
  v_r<-values(r)
  if (i==1){
    r_standard <- v_r
    raster_list<-stack(r)
  }else{
    raster_list<-addLayer(raster_list, r)
  }
  v_r <- v_r[which(!is.na(r_standard))]

  if (i==1){
    d_o<-data.frame(ID=c(1:length(v_r)))
  }
  d_o[,paste("V", i, sep="")] <- v_r
}



if (is_pca){
  # PCA
  pca <- prcomp(d_o[, c(2: dim(d_o)[2])],
                center = T,
                scale. = T)

  vars <- apply(pca$x, 2, var)
```

```
props_value <- vars / sum(vars)



current_proportion<-0
prop_index<-0
if (first_pcs==-1){
  while (current_proportion<=proportion_threshold){
    prop_index<-prop_index+1
    current_proportion<-current_proportion + props_value[prop_index]
  }
}else{
  prop_index<-first_pcs
  if (prop_index>length(props_value)){
    stop("first_pcs larger then number of variables.")
  }
}

# Generate PCs
p_d<-predict(pca,
             newdata=d_o[, c(2: dim(d_o)[2])])

sample_raster<-raster(raster_list, layer=1)

for (i in c(1:dim(p_d)[2])){
  #print(paste("Saving PC", i, "/", dim(p_d)[2]))
```

```
    values(sample_raster)<-NA
    values(sample_raster)[which(!is.na(r_standard))]<-p_d[,i]
    names(sample_raster)<-paste("PC", i, sep="")
    if (i==1){
      pc_list<-stack(sample_raster)
    }else{
      pc_list<-addLayer(pc_list, sample_raster)
    }
  }

  raster_list<-pc_list
}else{
  prop_index<-nlayers(raster_list)
}

prop_index=2

sp <- SpatialPoints(occ)
env_values<-extract(raster_list, sp)
env_values<-env_values[,1:prop_index]
dim(env_values)

train_d<-data.frame(occ, env_values)
#train_d<-train_d[!duplicated(train_d[,c(3:(2+prop_index))]),]

train_d[!complete.cases(train_d),]
```

```
env_values<-as.matrix(train_d[,c(3:(2+prop_index))])
plot(c(-4, 4), c(-6, 8), type="n",
     xlab="Axis 1",
     ylab="Axis 2" )
points(train_d$PC1, train_d$PC2)
mve_definition <- MVE_DEFINITION(env_values, 0.01)

ELLIPSEM(mve_definition[[2]][1:2,],mve_definition[[1]][1:2,1:2],
         npoints=100,c2=1,col='red', showcentre=F)



mve_definitions<-list(mve_definition)
vol<-VOLUME_MVE(mve_definition)
id<-1
vol_list<-data.frame(id=id, x=0, y=0, vol=vol)
old_train_d<-train_d
occurrences_threshold<-dim(train_d)[1]-max_removed_occurrences
if (occurrences_threshold<5){
  occurrences_threshold<-5
}

if (occurrences_threshold<=prop_index){
  occurrences_threshold<-prop_index+1
}
```

```
  while (dim(train_d)[1]>occurrences_threshold){

    print(paste("Occurrence size:", dim(train_d)[1]))

    min_vol<--1

    min_index<--1


    for (i in c(1:dim(train_d)[1])){

      print(dim(train_d)[1] - i)

      env_values<-as.matrix(train_d[,c(3:(2+prop_index))])

      env_values<-env_values[-i,]

      mve_definition <- MVE_DEFINITION(env_values, tolerance)

      vol<-VOLUME_MVE(mve_definition)

      if (min_vol==-1){

        min_vol<-vol

        min_index<-i

        min_mve_definition<-mve_definition

      }

      if (vol<min_vol){

        min_vol<-vol

        min_index<-i

        min_mve_definition<-mve_definition

      }


#Draw an ellipsoid in a plot
ELLIPSEM(min_mve_definition[[2]][1:2,],min_mve_definition[[1]][1:2,1:2],

               npoints=100,c2=1, col='red', showcentre=T)

    }
```

```
    id<-id+1
    mve_definitions[[id]]<-min_mve_definition
    vol_list<-rbind(vol_list,
                    data.frame(id=id, x=train_d[min_index, 1],
                               y=train_d[min_index, 2], vol=min_vol))
    print(paste("Remove No.", min_index))
    print(train_d[min_index,])

ELLIPSEM(min_mve_definition[[2]][1:2,],min_mve_definition[[1]][1:2,1:2],
             npoints=100,c2=1, col='blue', showcentre=T)
    train_d<-train_d[-min_index,]

  }
  result<-list(mve=mve_definitions, vol=vol_list)

  env_values<-as.matrix(old_train_d[,c(3:(2+prop_index))])

  mve_definition <- MVE_DEFINITION(env_values, 0.01)

  ELLIPSEM(mve_definition[[2]][1:2,],mve_definition[[1]][1:2,1:2],
           npoints=100,c2=1,col='black', showcentre=F)

  return(result)
}
```
