# Supplementary material for: Novel Methods in Disease Biogeography: A Case Study with Heterosporosis
Source: Front Vet Sci. 2017 Jul 17;4:105. doi: 10.3389/fvets.2017.00105 (PMC5511963; doi:10.3389/fvets.2017.00105)
Supplement: Supplementary Material S3 — R script to split the occurrence data for model evaluation as in Ref. (24) (TXT). [file data_sheet_3.pdf]

3 Supplementary Material S3

```
#Split occurrence data in two groups

#This is not a detailed explanation but just an example of the process employed to generate two independent sets of occurrences for calibration and evaluation.



setwd("C:/folder_name")# set working directory with the occurrences and environmental rasters.



# Install the R package ENMeval and the complemetary libraries (e.g. dismo, raster, rgdal).

library(ENMeval)#upload ENMeval.



occ<-read.table("occurrences_original.csv", head=T, sep=",")#upload the occurrences file in Maxent format (i.e., three columns: species, longitude, latitude).



occ<-occ[,c(2,3)]#Use information in columns 2 and 3.



env<-raster("Bio1.tif")#Upload one of the rasters that will be used for model calibration.



plot(env)#Display the raster

points(occ)#display the occurrences



#Split of occurrences

bg<-as.data.frame(env, xy=T)#Set the occurrences as data frame.

block_df<-get.block (occ, bg)#Split occurrences in block (see Muscarella et al. 2013. Ecography)
```

```
occ$group<-block_df$occ.grp#Asign occurrences into four groups (1, 2, 3, and 4).

#Display occurrences by group.

plot(occ$DecimalLongitude, occ$DecimalLatitude, pch=".", col=rainbow(7)[occ$group])

cal<-occ[which(occ$group %in% c(1,4)),]#Calibration occurrences from groups 1 and 4, i.e., off diagonal.

evl<-occ[which(occ$group %in% c(2,3)),]#Evaluation occurrences from groups 2 and 3, i.e., off diagonal.

#Save one group of occurrences as cal.csv

write.table(cal, "cal.csv", row.names = F, sep=",")#Save Calibration occurences as .csv file.

#Save one group of occurrences as evl.csv

write.table(evl, "evl.csv", row.names = F, sep=",")#Save Calibration occurences as .csv file.
```
