# Supplementary material for: Novel Methods in Disease Biogeography: A Case Study with Heterosporosis
Source: Front Vet Sci. 2017 Jul 17;4:105. doi: 10.3389/fvets.2017.00105 (PMC5511963; doi:10.3389/fvets.2017.00105)
Supplement: Supplementary Material S5 — Model evaluation results (DOCX). [file data_sheet_5.pdf]

4 Supplementary Material S5

. Model evaluation results. Evaluation of models calibrated with off-diagonal occurrences. Occurrences where split in four quadrants based on the longitude and latitude values as in (Peterson, 2012):

1	2
3	4

Occurrences from quadrants 1 and 4 where grouped and named **A**, while occurrences in quadrants 2 and 3 were groped and denoted **B**. Models were calibrated with **A** and evaluated later with **B** and vice versa. Evaluations were based on the number of independent points predicted correctly and the proportion of area predicted suitable by the models to estimate *p-values*:

		**All occurrences**		**Not outlier Occurrences**	
**Model**	**Algorithm**	**A vs B**	**B vs A**	**A vs B**	**B vs A**
N_F	MEV	5.76×10^{-3}	1.56×10^{-4}	1.56×10^{-3}	4.41×10^{-4}
N_R	Marble	5.33×10^{-15}	1.25×10^{-13}	4.15 × 10-4	2.64×10^{-9}
N_F	Maxent	5.80×10^{-8}	0.0	8.02 × 10-7	0.0
N_R	Maxent	0.0	$1.89 \text{x} 10^{-13}$	0.0	3.24×10^{-11}

N_F = Fundamental niche estimation. N_R = Realized niche estimation. MVE = Minimum-volume ellipsoid. A = occurrences of geographic quadrants 1 and 4. B = occurrences of geographic quadrants 2 and 3.
