# Supplementary figures and images for: Novel Methods in Disease Biogeography: A Case Study with Heterosporosis
Source: Front Vet Sci. 2017 Jul 17;4:105. doi: 10.3389/fvets.2017.00105 (PMC5511963; doi:10.3389/fvets.2017.00105)

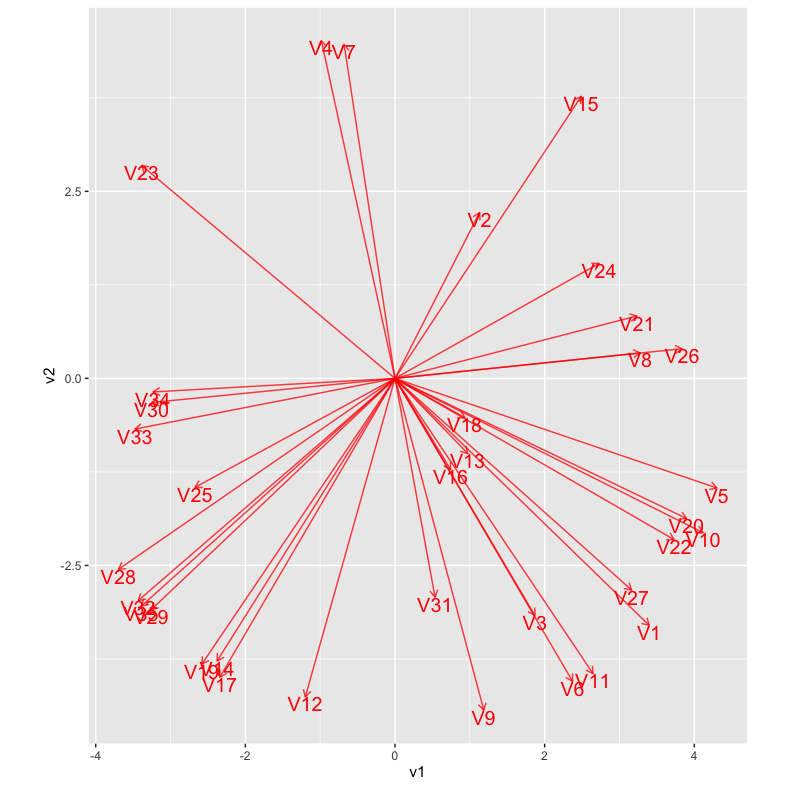

Supplement: Supplementary Material S6 — NicheA workflow and data to replicate analyses (XML). [file data_sheet_6.zip › Supplementary Material S6/background/biplot.png]

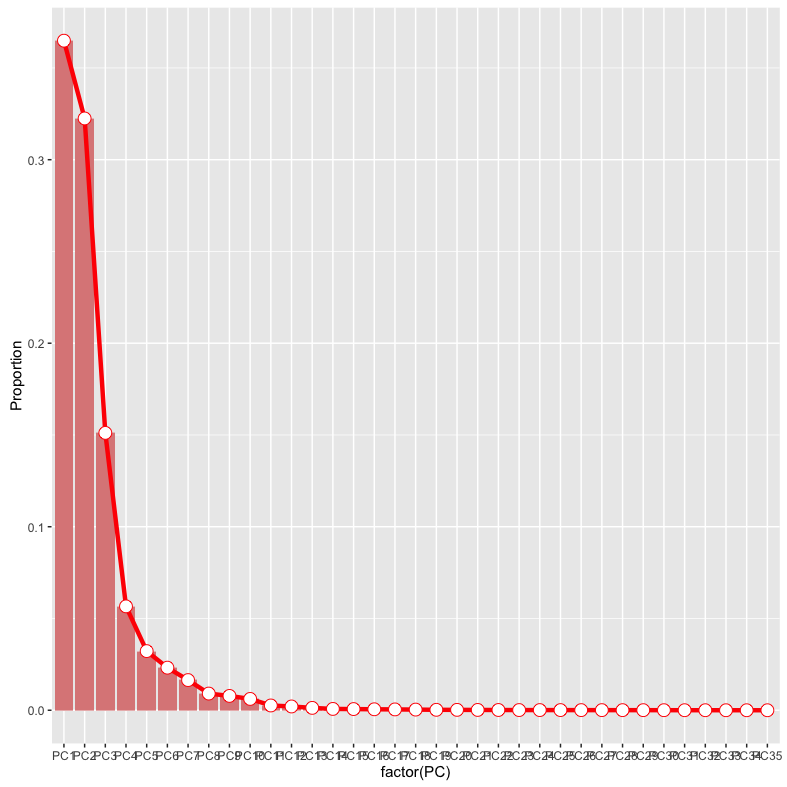

Supplement: Supplementary Material S6 — NicheA workflow and data to replicate analyses (XML). [file data_sheet_6.zip › Supplementary Material S6/background/proportion.png]
